# Nifedipine Upregulates ATF6-α, Caspases -12, -3, and -7 Implicating Lipotoxicity-Associated Renal ER Stress

**DOI:** 10.3390/ijms21093147

**Published:** 2020-04-29

**Authors:** Chiung-Chi Peng, Chang-Rong Chen, Chang-Yu Chen, Yen-Chung Lin, Kuan-Chou Chen, Robert Y. Peng

**Affiliations:** 1 Graduate Institute of Clinical Medicine, College of Medicine, Taipei Medical University, Taipei 11031, Taiwan; misspeng@tmu.edu.tw; 2 International Medical Doctor Program, Vita-Salute San Raffaele University, 20132 Milan, Italy; cherylcherylchen@gmail.com; 3 Program of Biomedical Sciences, College of Arts and Sciences, California Baptist University, Riverside, CA 92504, USA; eugenechen0529@gmail.com; 4 Division of Nephrology, Department of Internal Medicine, Taipei Medical University Hospital, Taipei 11031, Taiwan; yclin0229@tmu.edu.tw; 5 Division of Nephrology, Department of Internal Medicine, School of Medicine, College of Medicine, Taipei Medical University, Taipei 11031, Taiwan; 6 Department of Urology, School of Medicine, College of Medicine, Taipei Medical University, Taipei 11031, Taiwan; 7 Department of Urology, Taipei Medical University Shuang-Ho Hospital, Zhong-He District, New Taipei City 23561, Taiwan; 8 Department of Biotechnology, College of Medical and Health Care, Hungkuang University, Shalu District, Taichung 43302, Taiwan; ypeng@seed.net.tw

**Keywords:** chronic kidney disease, nifedipine, ER stress, ATF6α, lipotoxicity

## Abstract

Nifedipine (NF) is reported to have many beneficial effects in antihypertensive therapy. Recently, we found that NF induced lipid accumulation in renal tubular cells. Palmitic acid-induced renal lipotoxicity was found to be partially mediated by endoplasmic reticular (ER) stress, while it can also be elicited by NF in kidney cells; we examined the induction of suspected pathways in both in vitro and in vivo models. NRK52E cells cultured in high-glucose medium were treated with NF (30 µM) for 24–48 h. ER stress-induced lipotoxicity was explored by staining with thioflavin T and Nile red, transmission electron microscopy, terminal uridine nick-end labeling, and Western blotting. ER stress was also investigated in rats with induced chronic kidney disease (CKD) fed NF for four weeks. NF induced the production of unfolded protein aggregates, resulting in ER stress, as evidenced by the upregulation of glucose-regulated protein, 78 kDa (GRP78), activating transcription factor 6α (ATF6α), C/EBP-homologous protein (CHOP), and caspases-12, -3, and -7. In vitro early apoptosis was more predominant than late apoptosis. Most importantly, ATF6α was confirmed to play a unique role in NF-induced ER stress in both models. CKD patients with hypertension should not undergo NF therapy. In cases where it is required, alleviation of ER stress should be considered to avoid further damaging the kidneys.

## 1. Introduction

The endoplasmic reticulum (ER) is a cellular organelle involved in protein folding and secretion, calcium homeostasis, and lipid biosynthesis [1]. When ER homeostasis is disrupted by a variety of conditions, an adaptive mechanism known as the unfolded protein response (UPR) is activated to allow cells to cope with the pathophysiological agents/conditions known to elicit ER stress [1,2]. Glucose-regulating protein, 78 kDa (GRP78) is a master regulator of ER stress because of its role as a major ER chaperone regulating ER stress signaling pathways, leading to UPR survival and apoptosis responses [3]. The UPR is known to activate three distinct branches of signal transduction pathways, including protein kinase RNA-like ER kinase (PERK), inositol-requiring enzyme 1 (IRE1), and activating transcription factor 6 (ATF6) to restore ER proteostasis and regulate ER functions [4,5]. In cases where ER stress cannot be reversed, cellular functions deteriorate, often leading to cell death [1]. Protein misfolding in the ER often causes oxidative stress, which commits cells to apoptosis [6].

Evidence implies that ER stress-induced cellular dysfunction and cell death are major contributors to many diseases, including neurodegenerative diseases, diabetes, metabolic syndromes, cancers, hepatic dyslipidemia, steatosis, and chronic kidney disease (CKD) [1,7,8]. Upregulation of key UPR markers was observed in patients with primary glomerular disease [9,10,11] and diabetic nephropathy [12,13], and was strongly associated with proteinuria and renal fibrosis [14,15]. Inhibition of ER stress preserves glomerular barrier integrity and tubular function [8].

Nifedipine (NF), a known blocker of calcium channels, was reported to have many beneficial effects in addition to its antihypertensive action. NF inhibited high-glucose-induced caspase-3 activation and lamin B degradation in insulin-secreting INS-1 832/13 cells [16]. At a concentration of 33.3 mM, glucose-induced apoptosis in INS-1 β-cells via an ER stress pathway. NF inhibited Ca^2+^ release to protect β-cells from high-glucose-induced ER stress and apoptosis [17], implying that inhibition of Ca^2+^ over-accumulation provides a benefit of attenuating islet β-cell decompensation in a high-glucose environment [17]. Otherwise, 4-phenyl butyric acid (4-PBA), a known inhibitor of ER stress, markedly attenuated C/EBP homologous protein (CHOP) expression (an ER stress marker), caspase-3 activation, and lamin B degradation [16]. Nifedipine and diazoxide reduced palmitic acid-induced ER stress, exerting protective effects on pancreatic β-cells [18]. NF lowered the blood pressure and inhibited the development of glomerulosclerosis to the same extent as did moxonidine (a sympatholytic agent) in spontaneously hypertensive rats [19]. In addition, NF was also reported to exhibit many hypolipidemic effects, including enhancing lipolytic activity, accelerating clearance of postprandial lipemia [20], and inhibiting lipid peroxidation [21].

To our astonishment, recent work in our lab found that NF caused lipid accumulation and lipotoxicity in the kidneys [22], which showed controversial effects of NF against its other cited benefits [19,20,21]. Recently, it has emerged that the UPR can be directly activated by lipid perturbation, independently of misfolded proteins [23]. In reality, the lipotoxicity induced by palmitic acid in renal tubular cells was previously found to be partly mediated by ER stress [24]. To further investigate the possible mechanism of NF-induced renal lipotoxicity, this study examined the sequential induction of UPR-related pathways in both in vitro and in vivo models.

## 2. Results

### 2.1. NF Aggravated Renal Function in Rats with Doxorubicin (DR)-Induced CKD

Interestingly, NF aggravated the severity of DR-induced CKD in rats as evidenced by the excretion of large amounts of urinary proteins (248.4 ± 10.8 µg/dL) compared to 176.9 ± 13.2 µg/dL in the DR group and 66.3 ± 8.8 µg/dL in the control group (*p* < 0.05). However, the serum blood urea nitrogen (BUN)/creatinine ratios still remained rather comparable (Table 1).

### 2.2. NF Triggered Accumulation of Misfolded Proteins, Production of Reactive Oxygen Species (ROS), and Disruption of ER Folding

Thioflavins are dyes used for histological staining and biophysical studies of protein aggregation [25], and to visualize and quantify the presence of misfolded protein aggregates called amyloid (the same protein present in cerebral plaques of Alzheimer’s disease patients) [25]. This dye is also capable of detecting ER stress in living cells [26]. Production of ROS was correlated with ER stress and the UPR [6]. Dichlorofluorescein diacetate (DCFDA) is one of the most widely used techniques for directly measuring levels of intracellular ROS [27]. Thapsigargin (a positive control of ER stress) is a specific inhibitor of most animal intracellular sarcoplasmic/ER calcium ATPase (SERCA)-type Ca^2+^ pumps present in the sarcoplasmic/ER [28]. Thapsigargin inhibits ER Ca^2+^-dependent ATPase, leading to a depletion of ER Ca^2+^ storage, which in turn decreases the activities of Ca^2+^-dependent chaperones leading to an increase in unfolded proteins and a corresponding induction of UPR signaling [29].

**Figure 1:**
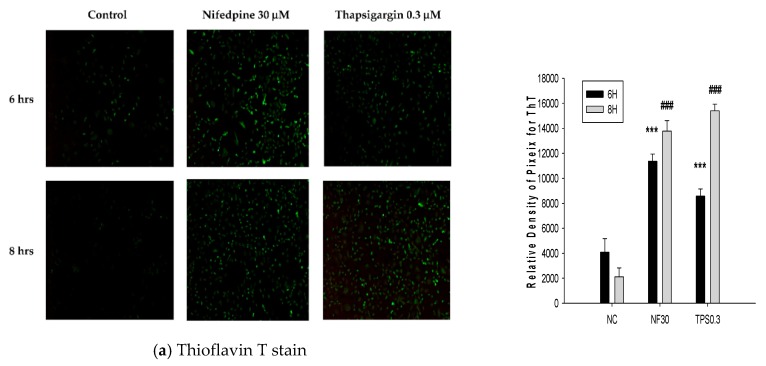

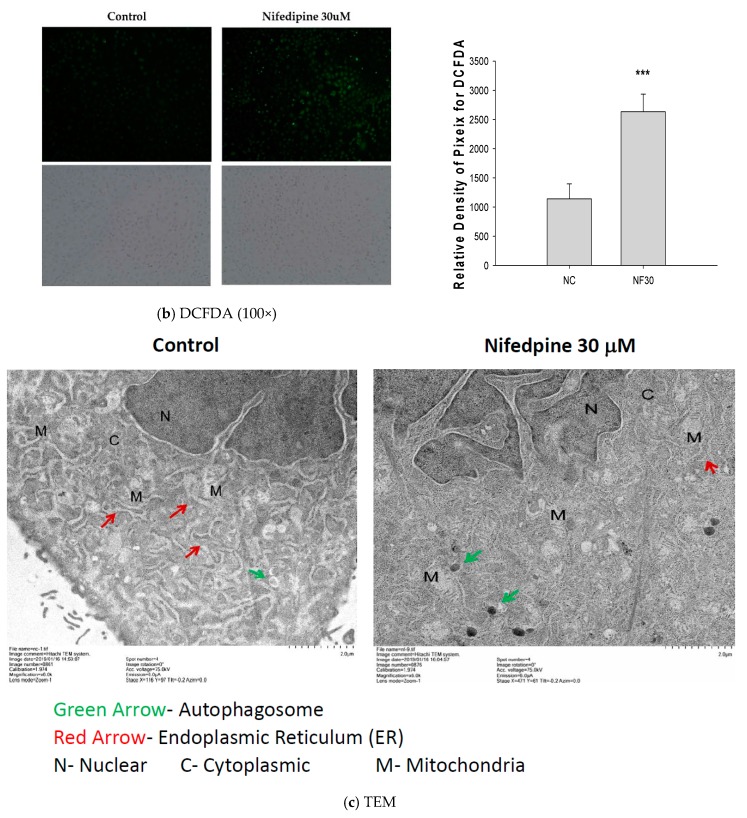
Nifedipine (NF) induced the ER stress and reactive oxygen species (ROS). (**a**) Representative images and quantitation of Thioflavin T staining for the detection of unfolded protein aggregates. Strong fluorescence appeared after treatment with NF (30 µM) for 6 and 8 h. Thapsigargin (TPS) (0.3 µM) was used as a positive control (100×). NC: normal control; NF30: nifedipine 30 µM; TPS0.3: thapsigargin 0.3 µM. * *P* < 0.1, ** *P* < 0.05, *** *P* < 0.01 compared to the normal control of 6 h. ^#^ *P* < 0.05, ^##^ *P* < 0.05, ^###^ *P* < 0.01 compared to the normal control of 8 h (*n* = 3). (**b**) Representative images and quantitation of DCFDA staining. The production of ROS induced by NF treatment (100×). *** *P* < 0.01 compared to the normal control (*n* = 3). (**c**) Transmission electron microscopy (TEM) showing a vast population of autophagosomes, and less endoplasmic reticular folding appeared after treatment with 30 µM NF (6000×).

After being treated with NF (30 µM) for 6 h, a vast number of large misfolded protein aggregates appeared in NRK52E cells as evidenced by the fluorescence intensity (Figure 1a, upper panel, middle). Similarly, treatment with 0.3 µM thapsigargin for 6 h produced tiny protein aggregates with less-strong fluorescence, compared to the negative control (Figure 1a). At 8 h after treatment, protein aggregates in thapsigargin-treated cells were seen to exhibit stronger fluorescence intensities (Figure 1a), implying a delayed reaction compared to that of NF. Furthermore, NF (30 µM) also obviously stimulated huge ROS production in NRK52E cells, as revealed by the appearance of intense DCFDA fluorescence (Figure 1b), and simultaneously, vast numbers of autophagosomes were observed under transmission electron microscopy (TEM) (Figure 1c).

### 2.3. UPR-ER Stress-Related Protein Signals Were Significantly Affected in NRK52E Cells In Vitro and Kidney Tissues In Vivo

**Figure 2:**
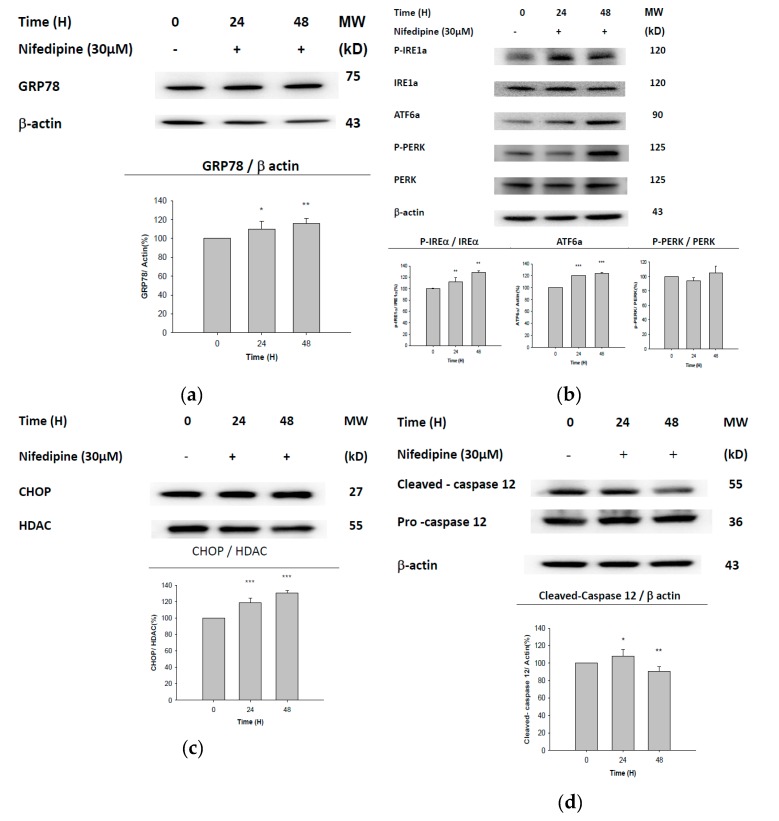
Representative Western blot and quantification of unfolded protein response (UPR)-endoplasmic reticular (ER) stress-related proteins elicited in NRK52E cells treated with nifedipine for 0–48 h. (**a**) Glucose-regulating protein, 78 kDa (GRP78); (**b**) phosphorylated (p)-inositol-requiring enzyme 1α (IRE1α), IRE1α, activating transcription factor 6α (ATF6α), phospho-protein kinase RNA-like ER kinase (PERK), PERK; (**c**) C/EBP homologous protein (CHOP); (**d**) procaspase-12, and cleaved caspase-12. β-actin and histone deacetylase (HDAC) were used as respective internal controls for the cytosolic and nuclear fractions. * *P* < 0.1, ** *P* < 0.05, *** *P* < 0.01 compared to 0 h (*n* = 3).

With 24–48 h of treatment with NF (30 µM), ER-associated proteins, including GRP78 (Figure 2a), phosphorylated (p)-inositol-requiring enzyme 1α (IRE1α), ATF6α (Figure 2b), and CHOP (Figure 2c), increased in NRK52E cells compared to control cells. In comparison, the expression of cleaved-caspase-12 increased from the beginning up to 24 h and then decreased afterward to below normal at 48 h (Figure 2d).

**Figure 3:**
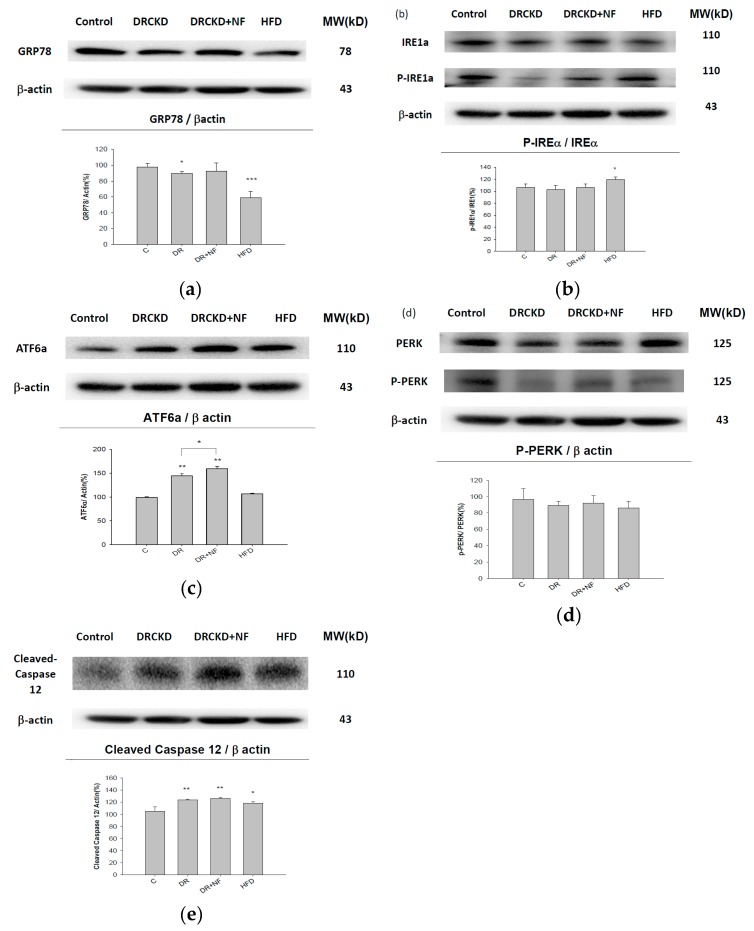
Representative Western blot and quantification of unfolded protein response (UPR)-endoplasmic reticular (ER) stress-related proteins in kidney tissues of rats with doxorubicin (DR)-induced chronic kidney disease. (**a**) Glucose-regulating protein, 78 kDa (GRP78), (**b**) inositol-requiring enzyme 1α (IRE1α), phosphorylated (p)-IRE1α, (**c**) activating transcription factor 6α (ATF6α), (**d**) protein kinase RNA-like ER kinase (PERK), p-PERK, and (**e**) cleaved caspase-12. β-actin was used as an internal protein control. Animal groups were treated with control (C), doxorubicin (DR), and DR and nifedipine (DR + NF), and fed a high-fat diet (HFD) * *P* < 0.1, ** *P* < 0.05, *** *P* < 0.01 compared to control group.

We found that the in vitro expression of GRP78 in kidney tissues was significantly downregulated in the DR and HFD groups (Figure 3a), and more prominently, there was no significant difference between the DR and DR + NF groups. Furthermore, the p-IREα/IREα ratio was only significantly higher in the HFD group, but not in the other groups (Figure 3b), while the p-PERK/PERK ratio was entirely unaffected in all groups, although PERK was significantly reduced in the DR and DR + NF groups (Figure 3d). Contrasting with those results, ATF6α was highly upregulated in the DR and DR + NF groups, but not in the HFD group, and more attractively, the ATF6α level in the DR + NF group was much higher than that in the DR group (Figure 3c). At the same time, levels of cleaved-caspase-12 were highly, but comparably, increased in kidney tissues of the DR and DR + NF groups, with a slightly but significantly lower level in the HFD group (Figure 3e).

Interestingly, HFD increased the expressions of p-IRE1α/IRE1α→caspase-12 in vivo, while GRP78 was downregulated (Figure 3b,e). Conversely, DR and DR + NF initiated ER stress via the ATF6α→caspase-12 pathway (Figure 3c,e).

### 2.4. ATF6α Was Confirmed to Be the Only ER Stress Pathway Affected by NF

**Figure 4:**
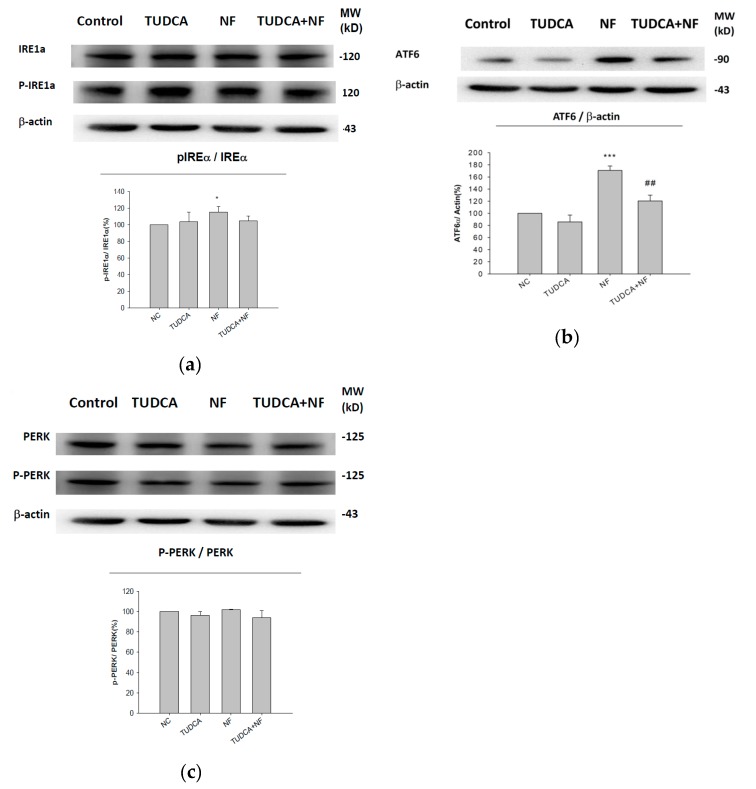
Protective effect of tauroursodeoxycholic acid (TUDCA) against nifedipine (NF) in NRK52E cells. Representative Western blots of (**a**) inositol-requiring enzyme 1α (IRE1α), phosphorylated (p)-IRE1α, (**b**) activating transcription factor 6α (ATF6α), (**c**) protein kinase RNA-like ER kinase (PERK), and p-PERK signal proteins. β-actin was used as the constitutive protein control. * *P* < 0.1, ** *P* < 0.05, *** *P* < 0.01 compared to normal control; ^##^ *P* < 0.05 compared to NF group (*n* = 3).

When ER stress was induced by NF, pretreatment with tauroursodeoxycholic acid (TUDCA; an ER stress inhibitor) was unable to significantly ameliorate expressions of p-IRE1α/IRE1α (Figure 4a) or p-PERK/PERK (Figure 4c), but only ATF6α, underscoring the relevant involvement of ATF6 in the UPR induced by NF (Figure 4b).

### 2.5. Early Apoptosis of NRK52E Cells Induced by NF May Partially Be Due to Activation of Caspase-3 and Caspase-7

**Figure 5:**
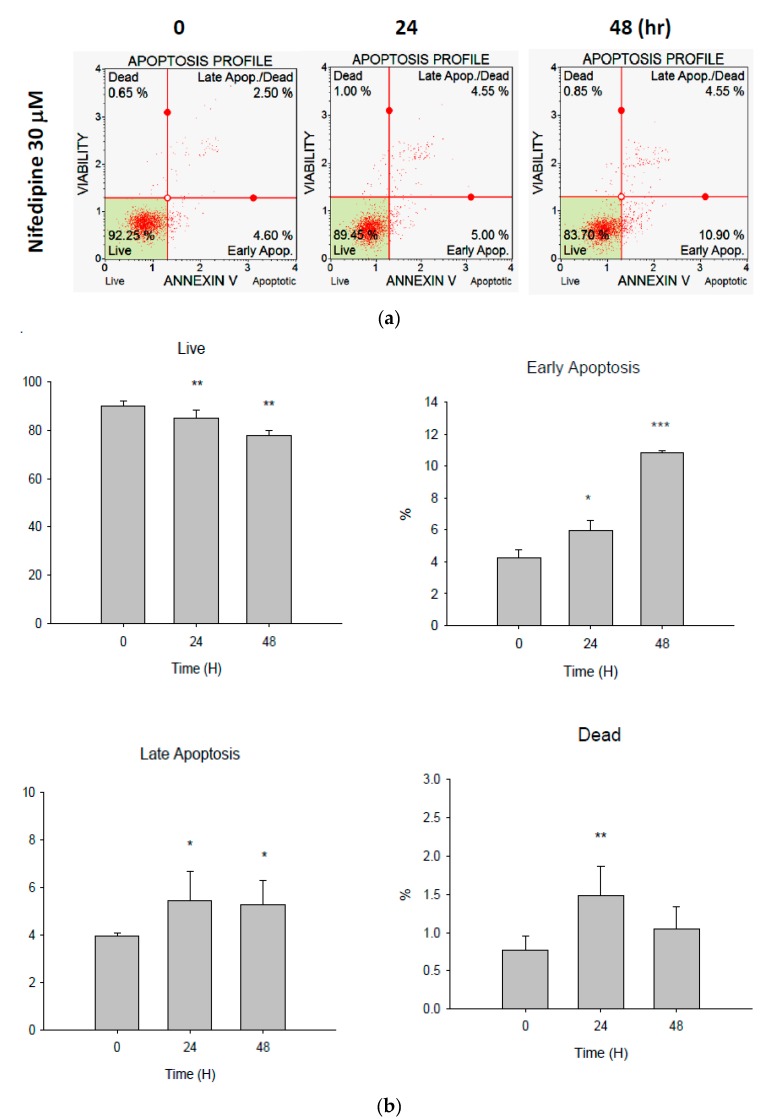

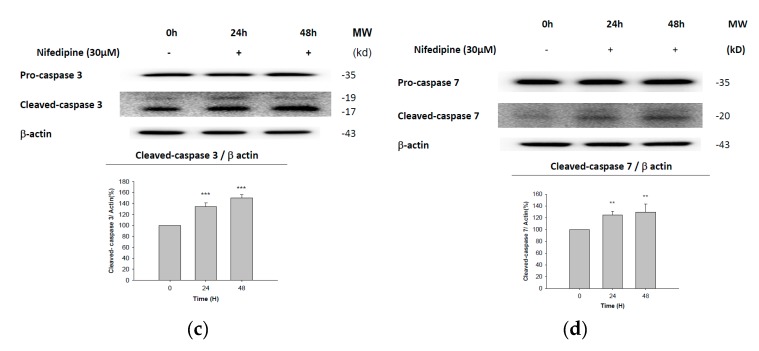
Cell apoptosis affected by nifedipine (NF) (30 µM). (**a**) Annexin V flow cytometric analysis. (**b**) Quantification of data of the Annexin V analysis. (**c**) Representative Western blots and quantitation of procaspase-3 and cleaved caspase-3 expressions. (**d**) Representative Western blots and quantitation of procaspase-7 and cleaved-caspase-7 expressions. β-actin was used as a constitutive protein control * *P* < 0.1, ** *P* < 0.05, *** *P* < 0.01 compared to 0 h (*n* = 3).

We found that after treatment with NF (30 µM) for 48 h, significant early apoptosis of NRK52E cells occurred from the initial 4.25% (at 0 h) and 5.96% (at 24 h) to 10.83% (at 48 h) (*p* < 0.05), contrasting with the occurrence of dead cells of 0.78% (at 0 h) and 1.48% (at 24 h) to 1.05% (at 48 h); and the occurrence of late apoptosis, of 3.97% (at 0 h) and 5.48% (at 24 h) to 5.28% (at 48 h) (*p* < 0.05) (Figure 5a,b). Cleaved caspase-3 and caspase-7 were both significantly upregulated at 24 h with further increases at 48 h (Figure 5c,d). Thus, upon activation of the UPR, caspase-12 may initiate the proapoptotic cascade involving caspase-3 and -7 activation in NF-treated NRK52E cells (Figure 2d and Figure 5c,d).

### 2.6. NF Also Enhanced Renal Apoptosis and Expression of Cleaved Caspase-3 in Rats with Experimentally Induced CKD

**Figure 6:**
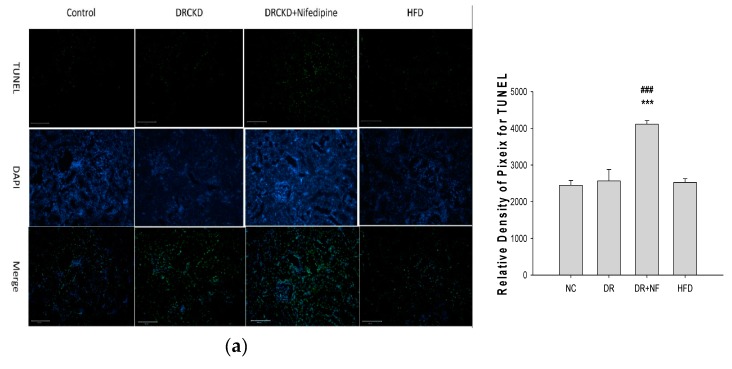

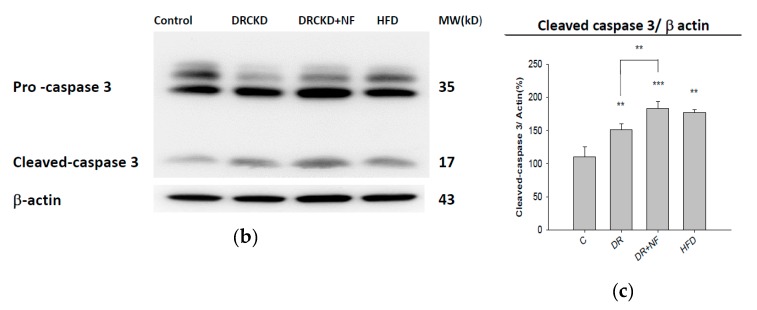
(**a**) Representative image and quantification for TUNEL staining of rat kidney tissues from the control, doxorubicin and chronic kidney disease (DRCKD), DRCKD + nifedipine (NF), and high-fat diet (HFD) groups. (**b**) Representative Western blot of pro-caspase 3 (35 kDa) and cleaved caspase 3 (17 kDa) of kidney tissues in rats as indicated. (**c**) Quantification of protein expression of cleaved caspase-3. β-actin was used as a constitutive protein control. Values are variations in percentage expressions relative to β-actin. ** *P* < 0.05, *** *P* < 0.01 compared to the normal control, ^##^ *P* < 0.05, ^###^ *P* < 0.01 compared to the DRCKD group (*n* = 3).

Populations of apoptotic cells in DR-induced rat kidneys were significantly upregulated compared to those of the control and HFD groups, which were further enhanced by co-therapy with NF as evidenced by a TUNEL assay (Figure 6a). The Western blot analysis of cleaved caspase-3 further confirmed such an event (Figure 6b,c). Cleaved caspase-3 was respectively upregulated to 151.3%, 182.7%, and 176.5% in the DR, DR + NF, and HFD groups, compared to 110.6% in the control group (Figure 6c).

### 2.7. Blockade of ER Stress Ameliorates Lipid Accumulation Induced by NF

**Figure 7:**
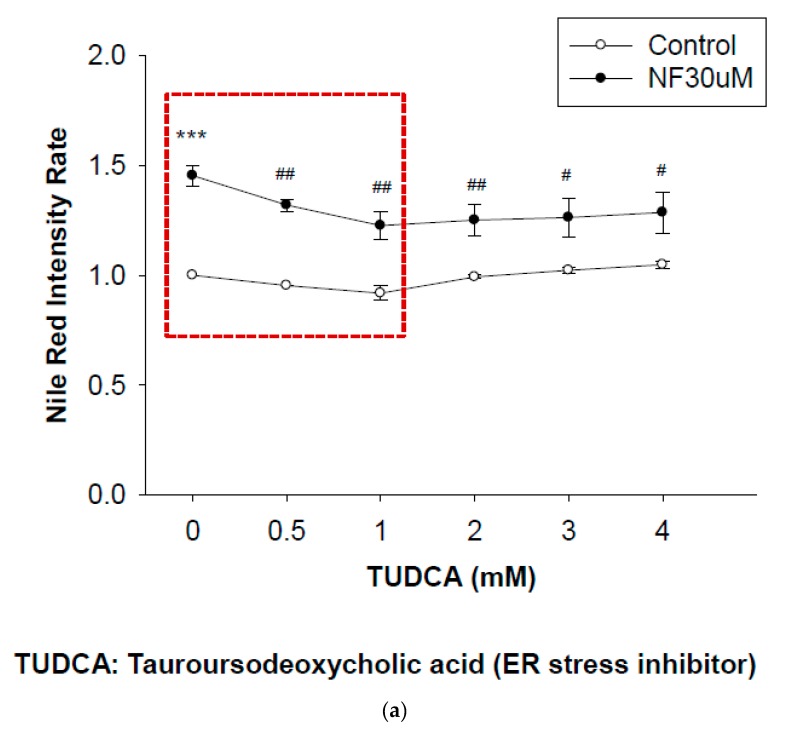

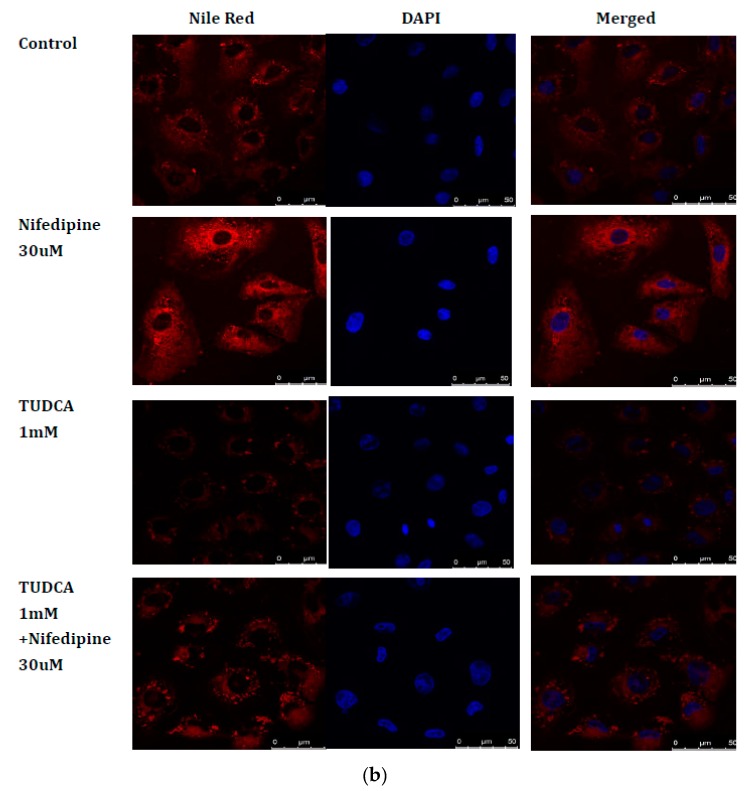
Detection of intracellular lipids in NRK52E cells by Nile red. (**a**) Flow cytometric analysis of the Nile red intensity affected by 30 µM of nifedipine (NF) with tauroursodeoxycholic acid (TUDCA) treatment. *** *P* < 0.01 compared to the control group only, ^#^ *P* < 0.05, ^##^ *P* < 0.05, ^###^ *P* < 0.01 compared to the NF only group (*n* = 3). (**b**) Confocal microscopic analysis of Nile red staining. First upper row, the controls. Second row, NF (30 µM) treatment. Third row, treatment with 1 mM of TUDCA. Bottom row, co-treatment with TUDCA (1 mM) and NF (30 µM).

As mentioned above, NF (30 µM) was shown to induce lipid accumulation in renal tubular cells [22]. More recently, we also found the accumulation of cholesterol after treatment with NF in an animal model (data not shown). Taken together, we suspected that the cellular UPR could be involved in such a lipotoxic phenomenon. TUDCA was applied prior to NF treatment. Data showed that TUDCA dose-dependently suppressed lipid accumulation until 1 mM, and then the response flattened off at doses of ≥1 mM (Figure 7a). The effect of TUDCA was more obvious in the NF-treated group than in the normal controls (Figure 7a). Lipid droplets were more densely distributed in the periphery of nuclei of NRK52E cells treated with NF, which was ameliorated when cells were pretreated with TUDCA (Figure 7b).

## 3. Discussion

Severe excretion of urinary proteins with comparably normal serum BUN/creatinine ratios (Table 1) may be attributed to the early stage of damage due to NF, because of an insufficiently long period for a positive response [30].

As data show in Figure 1a,b and Table 1, such results strongly urged us to suspect whether CKD damage caused by NF was associated with ER stress. The literature indicates that persistent protein misfolding might induce ROS cascades, leading to the progression of kidney disease [31]. Oxidative stress elicits the accumulation of advanced oxidation protein products and prompts the epithelial-to-mesenchymal transition (EMT) of renal tubular cells due to ER stress, resulting in accelerated progression of CKD [32]. To confirm this, we performed serial experiments with a cell model using NRK52E cells.

More attractively, TEM images revealed that ER structures were normally folded in the control, contrasting with those in the NF-treated groups, with the number of folded ER structures in the latter drastically decreasing (Figure 1c) [33]. Stoichiometrically, autophagosome formation can be triggered by ER stress as a secondary response when the quantity of misfolded proteins reaches or exceeds the ER capacity [1]. The consequences of autophagy might occur in duality, either being cytoprotective or cytotoxic to kidney cells depending on the cell type and pathological status [34].

Results in Figure 1 seem to imply that NF can induce ER stress. To verify this, the in vitro responses of UPR- and ER stress-related proteins were examined in parallel in an animal model (Figure 2 and Figure 3). As is well known, DR induces hypertension [35], while the hyperlipidemic status induced by an HFD model is well established using rats [36], and more pertinently, NF was reported to induce lipid accumulation in vitro [22]. Kidney tissues from the animal experiment were dissected and used to detect protein signals associated with ER stress (Figure 3). Much literature has emerged which shows that GRP78/BiP is critical for protein quality control of the ER as well as for activation of ER-transmembrane signaling molecules [3,37,38]. GRP78 acts as both a positive and negative regulator of the UPR by switching between receptors and unfolded proteins [39]; we propose such an effect can affect the progress of autophagy, an alternate phenomenon as described by Lhotak et al. (2012). Accumulated unfolded/misfolded proteins trigger the UPR in three transmembrane protein-mediated signaling pathways: the IRE1, PERK, and ATF6 pathways [40]. In the case of prolonged ER stress or UPR malfunction, apoptosis signaling is activated [41].

We previously demonstrated that NF induced ectopic lipid accumulation in rat NRK52E proximal tubular cells [22], and we suggested that this may involve the occurrence of ER stress (Figure 1). However, the major pathway involved in NF-induced UPR stress still remains unclear. In the present study, results from Western blotting, in both the in vitro and in vivo experiments, all uncompromisingly revealed the pathway via upregulation of ATF6α to caspase-12 to be a common phenomenon for UPR-ER stress induced by NF (Figure 2 and Figure 3).

ATF6, a member of the leucine zipper protein family which acts as a target of p38 mitogen-activated protein kinase (MAPK) phosphorylation [42], can constitutively induce the promoter of glucose-regulated protein (*grp*) genes through activation of the ER stress element (ERSE) [43]. It was demonstrated that ATF6 is translocated to the Golgi apparatus and proteolytically processed by sphingosine 1-phosphate (S1P) under ER stress. S1P also activates other ER stress-inducible transcription factors including sterol regulatory element-binding proteins (SREBPs) [44]. SREBPs play pivotal roles in de novo lipogenesis [45]. To further confirm if ATF6α is only true UPR signaling protein involved in NF-induced ER stress, NRK52E cells were pretreated with TUDCA before in vitro NF treatment (Figure 4). TUDCA is reported to protect against renal tubular injury in the presence of ER stress in diabetic *db/db* mice via decreasing ER stress-related apoptotic markers [46,47]. As mentioned above, NF not only induced SREBP expression but led to lipotoxicity in NRK52E cells [22]; thus ATF6 can be considered a chief ER transducer in response to NF-induced ER stress.

Annexin V is commonly used to detect apoptotic cells due to its ability to bind to phosphatidylserine, a marker of apoptosis when it is on the outer leaflet of plasma membranes. Elevated responses to Annexin V were found in both acute and chronic renal conditions [48]. Apoptosis of renal tubular epithelial cells is characterized by cell loss which plays a primary role in acute renal failure that contributes to CKD [49]. Obviously, in this regard, NF induced early apoptosis in NRK52E cells and in vivo by activating caspases-3 and -7 (Figure 5 and Figure 6). ER functions are very responsive to changes in extracellular-intracellular environments [50]. If the cellular UPR is persistent and stress cannot be resolved, signaling will switch from prosurvival to proapoptotic [51]. Palmitic acid induces the formation of intracellular lipid droplets and directly affects ER membrane proteins by promoting renal apoptosis in human proximal tubular HK2 cells [52].

Results shown in Figure 7 strongly imply the relevant association of ER stress with lipogenesis elicited in NRK52E cells and induced by NF. A similar study by Lhotak et al. found that when human kidney HK2 cells were treated with 10 μg/mL cyclosporine A (CsA), expressions of ER stress-related proteins and lipid accumulation prominently increased [53]. In addition, renal specimens from mice treated with tunicamycin (an ER stressor) and patients suffering from CsA-induced acute nephrotoxicity showed obvious lipid accumulation and ER stress in proximal tubules of the kidneys [53]. The accumulation of lipids in the kidneys is known as renal lipotoxicity according to Escasany et al. (2019), and it was reported to cause detrimental effects on the kidneys via several mechanisms of action, including reclusion of proinflammatory factors, development of oxidative and ER stresses, insulin resistance, deregulation of lipid metabolism, and overactivation of the renin–angiotensin aldosterone system [54].

To the present, however, one problem that has remained to be solved is whether NF induces lipotoxicity prior to ER stress or vice versa; obviously, further investigation is required.

## 4. Materials and Methods

### 4.1. Chemicals and Kits

NF (Sigma, St. Louis, MO, USA), thapsigargin (TPS) (Enzo Life Sciences, Farmingdale, NY, USA), tauroursodeoxycholic acid (TUDCA, Focus Biomolecules, Plymouth Meeting, PA, USA), thioflavin T (ThT) (ab120751, Abcam, Cambridge, MA, USA): 2′,7′-dichlorofluorescin diacetate (DCFDA) (Sigma-Aldrich, St. Louis, MO, USA), doxorubicin (DR) (Pfizer, Milano, Italy), and a high-fat diet (DIO rodent purified diet with 60% of the energy from fat, TestDiet, St. Louis, MO, USA) were purchased from various vendors. The protein extraction solution was from iNtRON Biotechnology (Burlington, MA, USA). All other chemicals unless otherwise stated were purchased from Sigma-Aldrich.

### 4.2. Cell Culture

The normal rat kidney epithelial-derived NRK52E cell line (CRL-1571) was purchased from the Bioresource Collection and Research Center (BCRC), Food Industry Research Development Institute (Hsinchu, Taiwan). Cells were cultured in 5% bovine calf serum-supplemented Dulbecco’s modified Eagle medium (DMEM) with 4 mM L-glutamine adjusted to contain 1.5 g/L sodium bicarbonate and 4.5 g/L glucose (Gibco, Carlsbad, CA, USA) at 37 °C in a humidified atmosphere with 5% CO_2_. Upon reaching 80% confluence, cells were trypsinized with 0.25% trypsin-0.02% ethylenediaminetetraacetic acid (EDTA) for 5 min at 37 °C and then re-passaged at a ratio of 1:3–1:4. NRK52E cells were treated with 30 µM NF for 24–48 h and then harvested for further analysis. The selected dose of NF corresponded to clinical use in humans [22,55,56,57]. Using a dose of 30 µM NF was based on the following reasons: (1) The clinically prescribed dose of 30–60 mg p.o. q.d., max 120 mg q.d. (FDA-approved recommended dose for treatment of hypertension) [58] corresponds to ca. 19–76 µM assuming a plasma volume of 4.5 L in males; and (2) to block 70% *I_Ca__(L)_*, 20 µM NF is required [59]. So, this dose amount was used in the study with the NRK-52E rat renal epithelial cell line [60,61].

### 4.3. Animal Experiments

This experiment was approved by the Institutional Animal Care and Ethics Committee of Taipei Medical University (Taipei, Taiwan). Briefly, 32 male Sprague-Dawley rats, aged 4 weeks and weighing 200–250 g, purchased from BioLasco (Taipei, Taiwan), were housed at three rats per cage and acclimated in the first week on basic chow in an animal room at 24 ± 2 °C, a relative humidity of 70–75%, and a 12/12-h light/night cycle. These rats were divided into four groups. Group 1 rats served as the normal control (Control, CTL). Each rat in group 2, the DR control (DRCKD), received a single shot of DR (7.5 mg/kg) intraperitoneally (IP) at the beginning or at week 2, and then rats in groups 1 and 2 were fed regular chow until the end of the experiment (week 9) with no further treatment. Each rat in group 3 (DRCKD + NF) received a single shot of DR as in group 2, and then also received an NF injection (0.5 mg/kg NF IP) beginning at the start of week 3, once daily from Monday to Friday each week and fed regular chow until week 9. To compare the lipidemic status in the kidneys [62], the chow of group 4 rats was changed to a high-fat diet (HFD) at the beginning of week 2, and those rats remained untreated until week 9. Blood and urine samples were collected at the end of weeks 1, 5, and 9. At the end of week 9, after collecting blood and urine samples, the rats were sacrificed, and kidney tissues were removed and rinsed with sterilized ice-cold phosphate-buffered saline (PBS) buffer, then stored at −20 °C. All samples were used for further experiments.

### 4.4. Western Blotting

NRK52E cells were treated with 30 µM NF as indicated. The extraction of cytoplasmic and nuclear proteins was carried out as instructed by the cytoplasmic and nuclear protein extraction kits (Biotools, New Taipei City, Taiwan). The proteins obtained were transferred to new Eppendorf flasks and stored at −80 °C. A standard protocol for Western blotting was used as described previously [35]. The specific primary antibodies used in this study included IRE1a, p-IRE1a, β-actin (Novus Biologicals, Littleton, CO, USA), PERK, p-PERK (Bioss Antibodies, Woburn, MA, USA), ATF6α (Santa Cruz Biotechnology, Santa Cruz, CA, USA), GRP78 (Epitomics, Abcam, Cambridge, MA, USA), CHOP, caspase-7, caspase-3 (Cell Signaling, Danvers, MA, USA), and caspase-12 (BioVision, Milpitas, CA, USA). Western blotting was repeated at least three times.

### 4.5. Thioflavin T (ThT) Stain

NRK52E cells were treated with NF were cultured on coverslips for the indicated times. Then, cells were stained with 3 µM of filtered ThT for 10 min at room temperature to detect protein aggregates. Coverslip slides were washed in 80% ethanol, >95% ethanol, and >distilled water and mounted in aqueous mounting media. Before analyzing the stained image under a fluorescent microscope (Olympus, Tokyo, Japan), the slides were dried in the dark overnight.

### 4.6. 2′,7′-Dichlorofluorescin Diacetate (DCFDA) Staining

In total, 2.5 × 10^4^ cells/well of NRK52E cells treated with or without NF were seeded in a 96-well plate and allowed to attach overnight. Cells were washed once in 1× PBS buffer, and then cells were stained with 25 µM DCFDA in 1× PBS buffer for 45 min at 37 °C. After removing all of the staining solution, the signal was monitored, and the sample was photographed at Ex/Em 485/535 nm with a fluorescent microscope (Olympus).

### 4.7. Transmission Electron Microscopy (TEM)

In total, 3 × 10^5^ NRK52E cells were seeded into two-well chamber slides and treated with 30 µM of NF for 16 h. Cells were fixed in 0.1 M cacodylate solution containing 2% paraformaldehyde and 2.5% glutaraldehyde for 1 h. After removing the fixative solution, cells were rinsed thrice with a buffer solution containing 0.1 M cacodylate and 7% sucrose, each time for 15 min. Specimens were stained with 1% OsO_4_ contrasting solution (in 0.1 M cacodylate buffer) for 1–2 h, and then subjected to a gradient ethanol dehydration method (i.e., 70% ethanol for 15 min; 80% ethanol for 15 min; 90% ethanol for 15 min; 95% ethanol twice, each time for 15 min; and finally 100% ethanol thrice, each time for 15 min). Dehydrated specimens were treated thrice with propylene oxide (PO), each time for 10 min. The resin had previously been evacuated under a vacuum for 8 h, and the PO/resin (1:1) was mixed and evacuated under a vacuum overnight. Specimens were embedded and heated to 62 °C in the oven for 48–72 h until completely set, then reshaped, sliced, and subjected to TEM HT7700 (Hitachi, Tokyo, Japan) analyses to access the intracellular ultrastructures.

### 4.8. Terminal Deoxynucleotidyl Transferase (TdT) dUTP Nick End Labeling (TUNEL) Assay

A TUNEL assay was carried out with an In Situ Cell Death Detection Kit (Roche Applied Science, Indianapolis, IN, USA). 4′,6-Diamidino-2-phenylindole (DAPI) counterstaining was conducted according to instructions given by the supplier, Thermo Fisher Scientific (Waltham, MA, USA). Results were examined under a fluorescent microscope (Olympus).

### 4.9. Nile Red Confocal Staining

Nile red (9-diethylamino-5H-benzo[alpha]phenoxazine-5-one) is an excellent vital stain for detecting intracellular lipid droplets by fluorescence microscopy and flow cytometry [63]. In this study, NRK52E cells treated with NF were stained with Nile red according to protocols of the manufacturer (Thermo Fisher Scientific, cat. no. N1142). Results were examined under the TCS SP5 Confocal Spectral Microscope Imaging System (Leica Geosystems, Heerbrugg, Switzerland).

### 4.10. Annexin V/Propidium Iodide (PI) Flow Cytometry

Annexin V/PI staining of NRK52E cells treated with or without NF was carried out under a Muse Cell Analyzer following protocols given by the Muse Annexin V and Dead Cell Kit (Merck-Millipore, Dresden, Germany).

### 4.11. Statistical Analysis

Statistical analyses were performed with Student’s *t*-test using SPSS 10.0 computer statistical software (SPSS, Chicago, IL, USA). An analysis of variance (ANOVA) was also used with Tukey’s test to analyze variances and the significance of the difference between paired means. The significance of the difference was judged by confidence levels of *^,#^ *p* < 0.1, **^,##^ *p* < 0.05, and ***^,###^ *p* < 0.01.

## 5. Conclusions

NF induces lipotoxicity in kidney cells, which can further damage kidneys that are progressing to CKD. In the present work, we found that NF induces extensive production of unfolded protein aggregates, resulting in ER stress and lipogenesis, while the main ER stress- and apoptosis-related marker proteins were all upregulated, including GRP78, ATF6α, cleaved caspase-12, CHOP, cleaved caspase-3, and cleaved caspase-7. These markers, in turn, led to more-significant early apoptosis than late apoptosis in NRK52E cells.

It is suggested that CKD patients with hypertension not be prescribed NF therapy. In cases where it is required, alleviating ER stress should be considered to avoid further damaging hypertensive kidneys.

## Figures and Tables

**Table 1:** Nifedipine (NF) aggravated the renal function of rats with doxorubicin (DR)-induced chronic kidney disease ^1^.

Group/Parameter	Control	DR	DR + NF
Urinary protein (µg/dL)	66.3 ± 8.8 ^a^	176.9 ± 13.2 ^b^	248.4 ± 10.8 ^c^
Serum blood urea nitrogen/creatinine	77.2 ± 5.4 ^a^	78.4 ± 10.0 ^a^	76. 3 ± 14.5 ^b^

^1^ Data are expressed as the mean ± standard deviation of triplicate experiments. ^a–c^ Values in each row with different lowercase superscripts significantly differ (*p* < 0.05). DR, doxorubicin-treated group; DR + NF, doxorubicin + nifedipine-treated group.

## Abbreviations

4-PBA	4-phenyl butyric acid
ATF6	activating transcription factor 6
CHOP	C/EBP homologous protein
CKD	chronic kidney disease
CsA	cyclosporine A
DCFDA	2′,7′-dichlorofluorescin diacetate
DR	doxorubicin
EMT	epithelial-to-mesenchymal transition
ER	endoplasmic reticulum
ERSE	ER stress element
HFD	high-fat diet
IRE1	inositol-requiring enzyme 1
NF	nifedipine
PBS	phosphate-buffered saline
PERK	PKR-like ER kinase
SREBP	sterol regulatory element-binding protein
TEM	transmission electron microscopy
ThT	thioflavin T
TPS	thapsigargin
TUDCA	tauroursodeoxycholic acid
TUNEL	terminal deoxynucleotidyl transferase (TdT) dUTP nick end labeling
UPR	unfolded protein response
